# Role of TDP2 in the repair of DNA damage induced by the radiomimetic drug Bleomycin

**DOI:** 10.1186/s41021-025-00329-9

**Published:** 2025-03-28

**Authors:** Naoto Shimizu, Kazuki Izawa, Mubasshir Washif, Ryosuke Morozumi, Kouji Hirota, Masataka Tsuda

**Affiliations:** 1 Program of Biomedical Science, Graduate School of Integrated Sciences for Life, Hiroshima University, Higashi-Hiroshima, Japan; 2 Division of Genome Safety Science, National Institute of Health Sciences, Kawasaki, Kanagawa Japan; 3 Department of Chemistry, Graduate School of Science, Tokyo Metropolitan University, Minamiosawa 1-1, Hachioji-shi, Tokyo, 192-0397 Japan; 4 Present address: Division of Cell-Based Therapeutic Products, National Institute of Health Sciences, Kawasaki, Kanagawa Japan

**Keywords:** Radiomimetic drugs, Bleomycin, Ionizing radiation, DNA double-strand breaks, TK6, Tyrosyl-DNA phosphodiesterase

## Abstract

**Background:**

Bleomycin (Bleo) is a glycopeptide with potent antitumor activity that induces DNA double-strand breaks (DSBs) through free radical generation, similar to ionizing radiation (IR). Therefore, Bleo is considered a radiomimetic drug. However, differences in DNA repair mechanisms between IR- and Bleo-induced DNA damage have not been fully elucidated. Therefore, in the present study, we examined a panel of repair-deficient human TK6 cell lines to elucidate the relative contributions of individual repair factors.

**Results:**

Our comprehensive profiling indicated that both non-homologous end joining (NHEJ) and homologous recombination (HR) contributed to DSB repair induced by X-rays and Bleo. Furthermore, tyrosyl-DNA phosphodiesterase (TDP)-related repair was a significant factor for cellular sensitivity to Bleo treatment. *TDP1*^*−/−*^*/TDP2*^*−/−*^ cells exhibited greater sensitivity to Bleo than *TDP1*^*−/−*^ or *TDP2*^*−/−*^ cells, but not to X-rays. In addition, we determined whether TDP2 is involved in the repair of Bleo-induced DSBs using a neutral comet assay. In TDP1-deficient cells, knockout of *TDP2* resulted in a significant delay in the repair kinetics of DSBs induced by Bleo, but not by X-rays.

**Conclusions:**

The contribution of the TDP-related pathway to DSB repair significantly differed between IR and radiomimetic drugs. The discovery of this novel TDP2-dependent repair of DSBs resulting from radiomimetic drug exposure indicates that TDP1 and TDP2 inhibition in combination with radiomimetic drugs represents a strategy for cancer treatment.

**Supplementary Information:**

The online version contains supplementary material available at 10.1186/s41021-025-00329-9.

## Background

Bleomycin (Bleo) is a glycopeptide that exhibits potent antitumor activity against lymphomas; malignant germ cell tumors; and carcinomas of the skin, head, and neck [1, 2]. Bleo generates free radicals in DNA, which results in DNA double-strand breaks (DSBs) with a biological effect similar to those induced by ionizing radiation (IR) [3]. Bleo induces DSBs and single-strand breaks (SSBs) in a ratio of approximately 1:6‒1:20 [4, 5], whereas IR exposure causes DSBs and SSBs in a ratio of approximately 1:20. Bleo is a radiomimetic drug; however, sensitivity to Bleo is not necessarily correlated with radiation sensitivity in head and neck squamous cell lines [6]. One possible explanation for this phenomenon is that different DNA repair pathways may operate in response to DNA damage induced by IR and Bleo.

It is well established that both IR and Bleo induce DNA damage, and the differences in the lesions they produce have been extensively characterized. IR deposits energy into molecules, leading to the cleavage of chemical bonds; as it traverses cells, it disrupts covalent bonds within genomic DNA. Consequently, IR induces a range of DNA lesions, including base modifications, SSBs, DSBs, and DNA-protein crosslinks (DPCs) [7]. In contrast, Bleo becomes activated upon binding to Fe(II), followed by the binding of oxygen and reduction by a reductant. This activated Bleo induces DSBs with characteristic 3′-phosphoglycolate (3′-PG) and 5′-phosphate ends and also generates 4′-oxidized abasic sites, where the base is lost and the sugar backbone is altered [5]. These lesions may disrupt essential cellular processes such as replication and transcription. Thus, although IR and Bleo induce distinct spectra of DNA damage, it remains unclear how these differences affect the mechanisms that repair DNA damage induced by each agent.

The DSB repair mechanism has primarily been elucidated using IR [7]. Eukaryotic cells employ two major DSB repair pathways that play a significant role in preventing cell death: homologous recombination (HR) and nonhomologous end joining (NHEJ) [8, 9]. HR involves several steps, including end resection on the DSB strand, homology search, strand invasion into the homologous template, and DNA repair synthesis [10]. RAD54 is an important protein that promotes strand invasion during HR [11]. In NHEJ, the broken ends of two DSBs are directly rejoined without the use of homologous templates. The Ku heterodimer initiates the process, which results in the activation of the DNA-dependent protein kinase catalytic subunit (DNA-PKcs) and recruitment of DNA ligase 4 (LIG4) to seal the broken ends [12]. During the G1 phase, 53BP1 promotes NHEJ [13]. The base damage is converted into an SSB (DNA nick) as an intermediate step in the repair process [14]. With a DNA nick, poly (ADP-ribose) polymerase 1 (PARP-1) gets activated and poly (ADP-ribosyl) ates several proteins, including itself [15, 16]. PARP-1 predominantly interacts with the X-ray repair cross-complementing group 1 (XRCC1) protein [17, 18]. At the DNA damage site, XRCC1 contributes to repair by recruiting DNA polymerase (POL) β, which is involved in gap-filling synthesis and removal of the 5′-blocking group [19, 20]. The repair of DPCs induced by formaldehyde and etoposide is mediated by the metalloprotease SPRTN [21]. The repair of DPCs induced by topoisomerase inhibitors and formaldehyde is initiated not only by SPRTN but also by the proteasome [19]. Notably, *SPRTN*^*−/−*^ cells are not sensitive to IR, suggesting that SPRTN does not participate in the repair of IR-induced DPCs [22]; however, the overall differences in the contributions of these factors to the repair of DNA damage induced by IR and Bleo remains unclear.

A set of isogenic human TK6 cells that are DNA repair mutant clones and possess functional p53 comparable to normal human tissues exhibiting rapid proliferation (13 h/cell cycle) has been established [23]. Using this mutant panel, the role of individual repair pathways following IR irradiation in cell survival was examined between normoxic and hypoxic cells [22]. Recently, we used comprehensive profiling to demonstrate that the tyrosyl-DNA phosphodiesterase (TDP)-related repair pathway is a primary contributor to the repair of DNA damage induced by Camptothecin (CPT) [24].

In the present study, we compared the sensitivity of a panel of DNA repair-deficient TK6 cell lines to X-rays and Bleo. Distinct sensitivity patterns were observed among TDP-related repair mutant cells. Interestingly, *TDP1*^*−/−*^*/TDP2*^*−/−*^ cells exhibited increased sensitivity to Bleo, but not to X-rays, compared with single mutant cells. The absence of TDP2 enhanced the delay in DSB repair in *TDP1*^*−/−*^ cells following treatment. These findings suggest a novel role of TDP2 in DSB repair induced by Bleo.

## Materials and methods

### Cell culture

Human lymphoblast TK6 cells [25] were supplied by Dr. Shunichi Takeda and Dr. Hiroyuki Sasanuma (Department of Radiation Genetics, Graduate School of Medicine, Kyoto University; Table 1). The cells were cultured in RPMI-1640 medium (189–02025, Wako, Osaka, Japan) supplemented with 5% heat-inactivated horse serum, L-glutamine (16948-04, Nacalai Tesque, Kyoto, Japan), 0.2 mg/mL sodium pyruvate (P2256, Sigma-Aldrich, Steinheim, Germany), 100 U/mL penicillin, and 100 µg/mL streptomycin (168–23191, Nacalai Tesque, Kyoto, Japan) and maintained at 37 °C in a humidified atmosphere containing 5% CO_2_ as previously described [22, 26].

### Cell survival

X-rays were generated by an OHMic OM-303 M X-ray generator (70 kV, 3 mA, 0.2 mm Al filter). Bleomycin sulfate (Bleo; B3972) was purchased from TOKYO CHEMICAL INDUSTRY (Tokyo, Japan). Cells were either irradiated with X-rays or treated with Bleo for 3 h at 37 °C. The cells were seeded in triplicate in six-well plates with 5 mL/well of 1.5% (w/v) methylcellulose (M0387, Sigma-Aldrich, Steinheim, Germany) and D-MEM/Ham’s F-12 (042–30555, Wako, Osaka, Japan) supplemented with 10% horse serum. The number of colonies was counted at day 10 to 14 [26–28].

### Neutral comet assays

Cells were either irradiated with 20 Gy X-rays or treated with 400 µg/mL Bleo for 30 min at 37 °C, and incubated in drug-free media for 180 min. The cells were embedded in agarose, treated with lysis buffer, and electrophoresed as previously described [24]. The slides were observed under a fluorescence microscope (TE2000; Nikon, Tokyo, Japan) at 200× magnification. OpenComet software was used to measure the tail moments from 50 cells/sample [29].

### Statical analysis

Significant differences were identified using Tukey’s multiple comparison test, ANOVA, and Student’s t-test implemented in scipy (1.6.2).

### Biochemical analysis of 3′-PG processing

The 3′-PG oligonucleotide (5′-TCCCCAACTAACATGAACTCGACG) was purchased from Eurogentec (Seraing, Belgium). The 3′-PG oligonucleotide was 5′-labeled with ^32^P using [γ-^32^P] ATP and T4 polynucleotide kinase (2021 S, TAKARA). The labeled DNA substrates were incubated with the indicated concentrations of recombinant human TDP1 (ab131921, Abcam, Cambridge, UK) or TDP2 (#TG2003H, TopoGEN, Co, USA) for 30 min at 37 °C in 5 µl of reaction buffer. Reactions were terminated by adding one volume of gel loading buffer (formamide containing 2.5 mM EDTA). Samples were separated using 20% denaturing polyacrylamide gel containing 7 M urea in TBE buffer (89 mM Tris, 89 mM boric acid, and 2 mM EDTA). Following electrophoresis, the radioactivity of the gel was measured using a Typhoon FLA9500 (GE Healthcare Life Sciences).

## Results

### Contribution of TDP-dependent repair to Bleo-induced DNA damage response

**Fig. 1:**
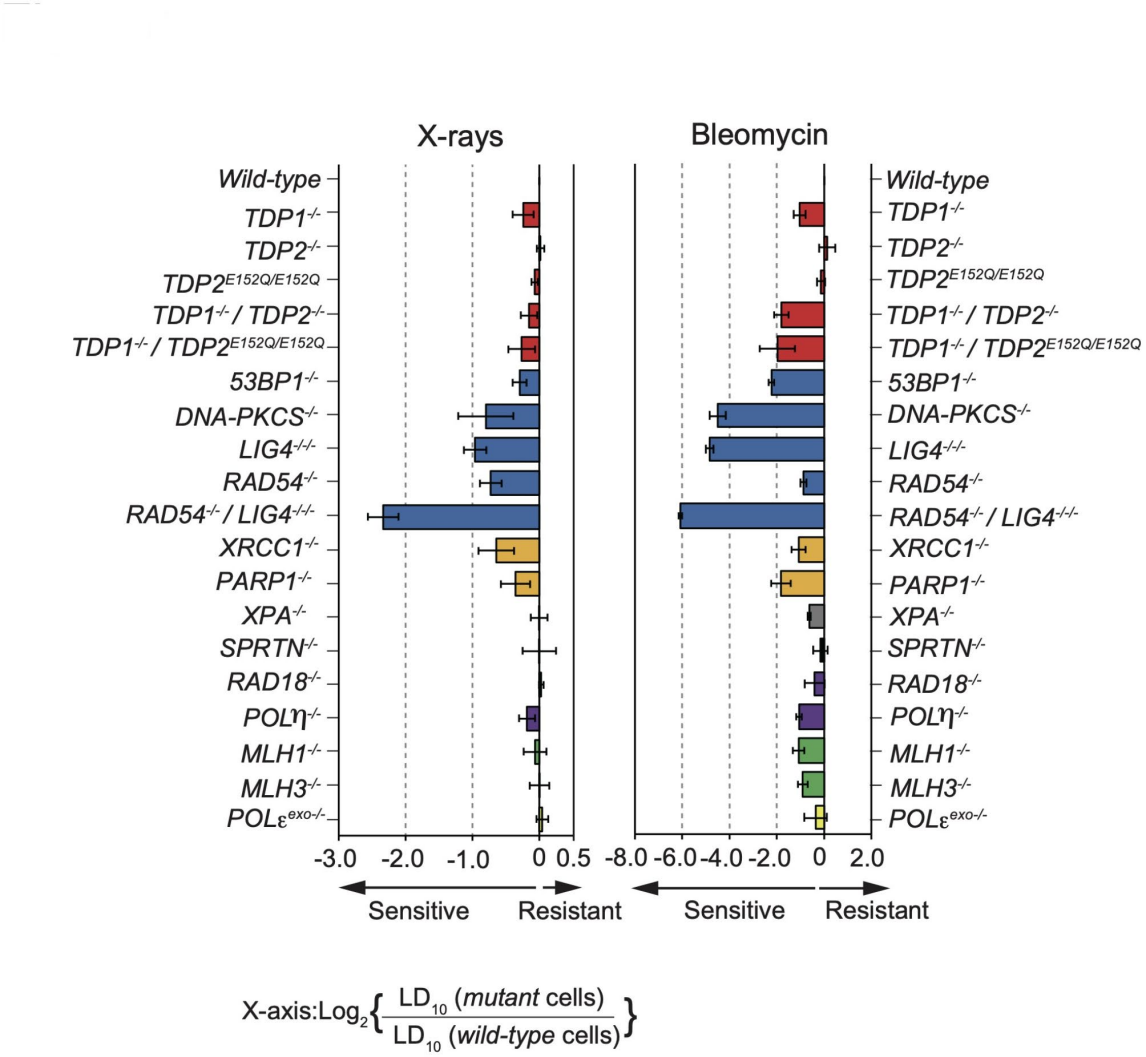
X-rays and Bleo sensitivity profiles of selected DNA repair-deficient TK6 cells. The sensitivity of the mutant cells relative to *wild-type* cells was determined as described in the Materials and Methods. Negative and positive scores indicate the sensitivity and resistance of the given cell lines, respectively. Relative sensitivity was computed as follows: Log_2_ [(LD_10_ in mutant cells) / (LD_10_ in *wild-type* cells)]. Each bar is colored based on the DNA repair function category: red, TDP-related repair; blue, DSB repair; orange, base excision repair (BER); gray, nucleotide excision repair (NER); black, DPC repair; purple, translesion synthesis (TLS); green, mismatch repair (MMR); and yellow, proofreading of replicative polymerase. Error bars indicate standard deviations of the mean of three independent assays

The sensitivity of a panel of DNA repair-deficient TK6 cell lines, consisting of 19 mutant cell lines, was examined using X-rays and Bleo (Table 1). The doses resulting in 10% survival (LD10) were calculated. Figure 1 shows the ratio of LD10 values between individual isogenic mutants and *wild-type* cells on a logarithmic scale. Our findings revealed that NHEJ-deficient (*LIG4*^*−/−/−*^, *DNA-PKCS*^*−/−*^, and *53BP1*^*−/−*^) cells were sensitive to both X-rays [22] and Bleo. In contrast, HR deficiency resulted in hypersensitivity to X-rays but minimal sensitivity to Bleo. These findings suggest that while both NHEJ and HR contribute equally to X-ray-induced DSB repair, NHEJ plays a more prominent role in repairing DSBs induced by Bleo compared with HR. Furthermore, *RAD54*^*−/−*^*/LIG4*^*−/−/−*^ cells exhibited greater sensitivity to X-rays and Bleo than *RAD54*^*−/−*^ or *LIG4*^*−/−/−*^ cells. Thus, DSBs are the major type of DNA damage induced by both X-rays and Bleo. Overall, the sensitivity profiles of these agents were similar (Fig. 1). Interestingly, TDP-related mutant cells displayed distinct sensitivity profiles between X-rays and Bleo. While *TDP1*^*−/−*^ cells were sensitive to Bleo, they exhibited little sensitivity to X-rays. In addition, *TDP1*^*−/−*^*/TDP2*^*−/−*^ cells were more sensitive to Bleo than single mutant cells. The Bleo sensitivity of TDP-related repair mutant cells is similar to CPT sensitivity of the cells [24, 30]. CPT is a well-known anticancer agent that inhibits DNA topoisomerase 1 (TOP1), resulting in the trapping of TOP1-DNA cleavage complexes (TOP1ccs) [31, 32] that are repaired by TDP1 [33]. TDP2 primarily repairs trapped TOP2-DNA cleavage complexes (TOP2ccs) [34], and TDP2 repairs TOP1ccs in the absence of TDP1 [30, 35]. Therefore, TDP2 appears to be involved in the repair of Bleo and CPT-induced DNA damage in the absence of TDP1.

**Table 1:** Panel of cell lines used in this study

Genotype	Functions of the deleted gene(s)	References
*TDP1* ^*−/−*^	TDP-related repair	[36]
*TDP2* ^*−/−*^	TDP-related repair	[34, 36]
*TDP2* ^*E152Q/E152Q*^	TDP-related repair	[24]
*TDP1* ^*−/−*^ */TDP2* ^*−/−*^	TDP-related repair	[30, 37]
*TDP1* ^*−/−*^ */TDP2* ^*E152Q/E152Q*^	TDP-related repair	[24]
*53BP1* ^*−/−*^	DSB repair (NHEJ)	[38]
*DNA-PKCS* ^*−/−*^	DSB repair (NHEJ)	[39]
*LIG4* ^*−/−/−*^	DSB repair (NHEJ)	[39]
*RAD54* ^*−/−*^	DSB repair (HR)	[39]
*RAD54* ^*−/−*^ */LIG4* ^*−/−/−*^	DSB repair (HR/NHEJ)	[39]
*XRCC1* ^*−/−*^	BER	[40]
*PARP1* ^*−/−*^	BER	[37]
*XPA* ^*−/−*^	NER	[26]
*SPRTN* ^*−/−*^	DPC repair, TLS	[41]
*RAD18* ^*−/−*^	TLS	[28]
*POLη* ^*−/−*^	TLS	[26]
*MLH1* ^*−/−*^	MMR	[42]
*MLH3* ^*−/−*^	MMR	[42]
*POLε* ^*exo−/−*^	Proofreading of replicative polymerase	[28]

*BER* base excision repair; *DPC* DNA-protein crosslink; *HR* homologous recombination; *MMR* mismatch repair; *NER* nucleotide excision repair; *NHEJ* nonhomologous end joining; *TDP* tyrosyl-DNA phosphodiesterase; *TLS* translesion DNA synthesis

We previously identified the critical Glu152 residue in TDP2, which is responsible for binding and stabilizing the catalytically essential Mg^2+^ required for repairing both trapped TOP1ccs and TOP2ccs [24]. In TDP1-deficient human TK6 cells, but not *wild-type* cells, a Glu152Gln alteration in both alleles of *TDP2* resulted in a significant increase in Bleo sensitivity, whereas X-ray sensitivity was unaffected (Fig. 1). These data indicated the important role of Mg^2+^ binding to TDP2 in the repair of not only trapped topoisomerases but also Bleo-induced DNA damage.

### Assessment of DSB repair kinetics in TDP-related mutant cells following X-ray exposure

**Fig. 2:**
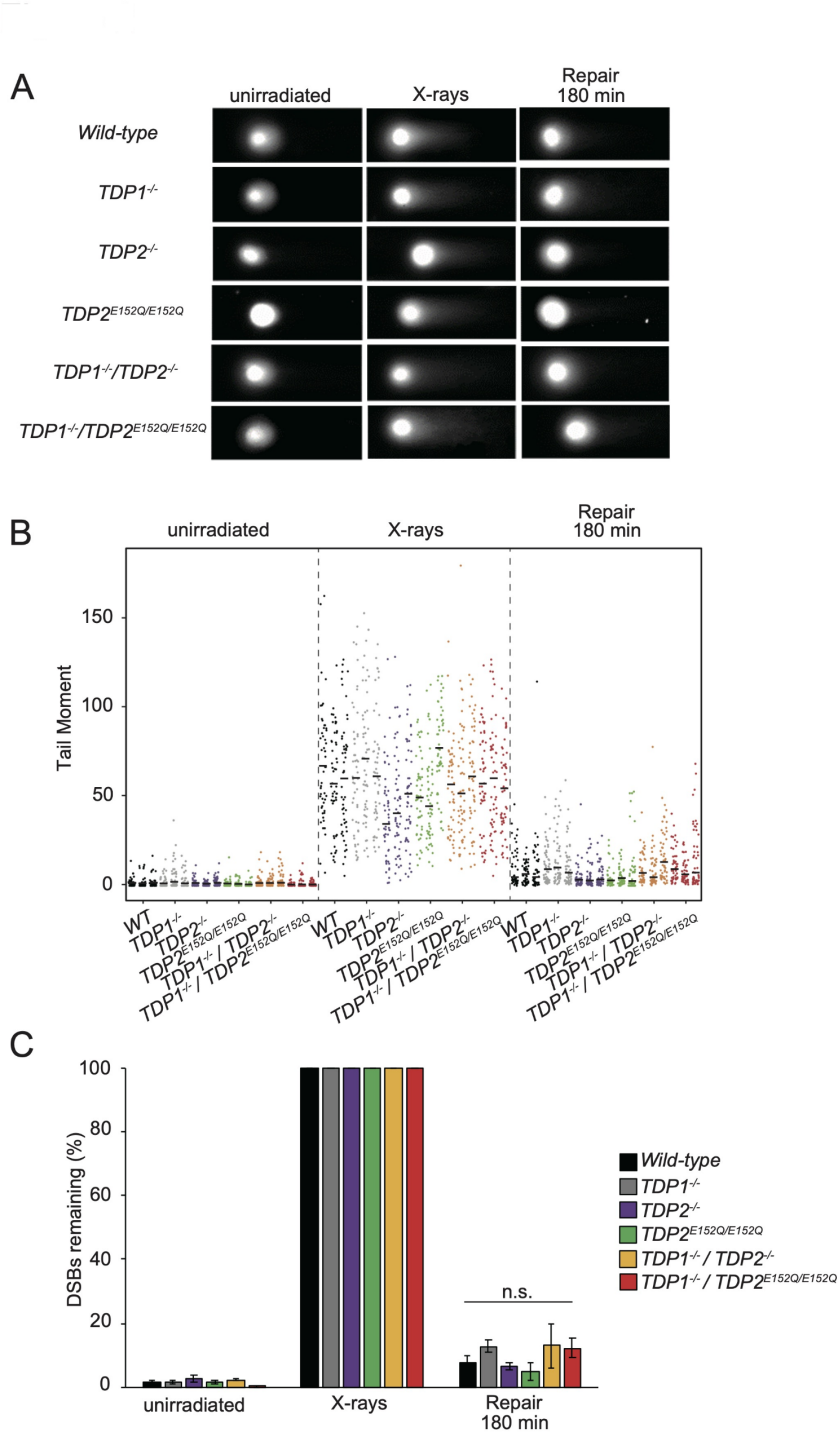
Repair kinetics of DSBs in X-ray-irradiated cells. **A** Typical neutral comet images. The indicated cells were irradiated with and without X-rays, incubated in medium for 180 min, and analyzed for DSBs using neutral comet assays. The images below the X-rays show the samples prepared immediately after X-ray irradiation (0 min repair). **B** Tail moments (raw data) of irradiated cells in neutral comet assays. In total, we assessed 50 cells/sample and conducted experiments in triplicate for each cell type. Tail moments of different cells of each cell type from three experiments are plotted vertically in three separate columns. Lines indicate median tail moments. **C** Median tail moments were quantified for 50 cells/sample/experiment and standardized to those after 0 min of repair, bars on X-rays. DSBs remaining are presented as a percentage of the remaining damage. Error bars are standard deviations of the mean of three independent assays. Significant differences were identified using an ANOVA: n.s. = not significant. Median tail moments before standardization are shown in Fig. S1A

We hypothesized that TDP2 is specifically involved in a novel repair pathway for DSBs induced by Bleo, not by X-rays, in the absence of TDP1. The kinetics of DSB repair can be monitored using a neutral comet assay [43]. To investigate DSB repair kinetics, we irradiated *wild-type*, *TDP1*^*−/−*^, *TDP2*^*−/−*^, *TDP2*^*E152Q/E152Q*^, *TDP1*^*−/−*^*/TDP2*^*−/−*^, and *TDP1*^*−/−*^*/TDP2*^*E152Q/E152Q*^ cells. These cells were then allowed to recover in a drug-free medium for 180 min. Figure 2A shows typical images from a neutral comet assay. We analyzed 50 cell images per sample and plotted individual comet tail moments (an arbitrary measure of DSB). In all cell types, X-ray exposure caused an increase in the tail moment (Fig. 2B). Due to the observed variation in tail moments among mutants following X-ray exposure (Supplementary Table 1, Fig. S1A), we applied data standardization for the analysis of DSB repair capacity (Fig. 2C). At 180 min after X-ray exposure, the remaining DSBs were similar among *wild-type*, *TDP1*^*−/−*^, *TDP1*^*−/−*^*/TDP2*^*−/−*^, and *TDP1*^*−/−*^*/TDP2*^*E152Q/E152Q*^ cells (*p* > 0.005, ANOVA), indicating that TDP2 knockout in *TDP1*^*−/−*^ cells did not significantly affect DSB repair kinetics. These results indicate that TDP1 and TDP2 do not significantly contribute to the repair of DSB caused by X-ray exposure.

### Delayed DSB repair kinetics of *TDP1*^*−/−*^*/TDP2*^*−/−*^ cells following Bleo treatment

**Fig. 3:**
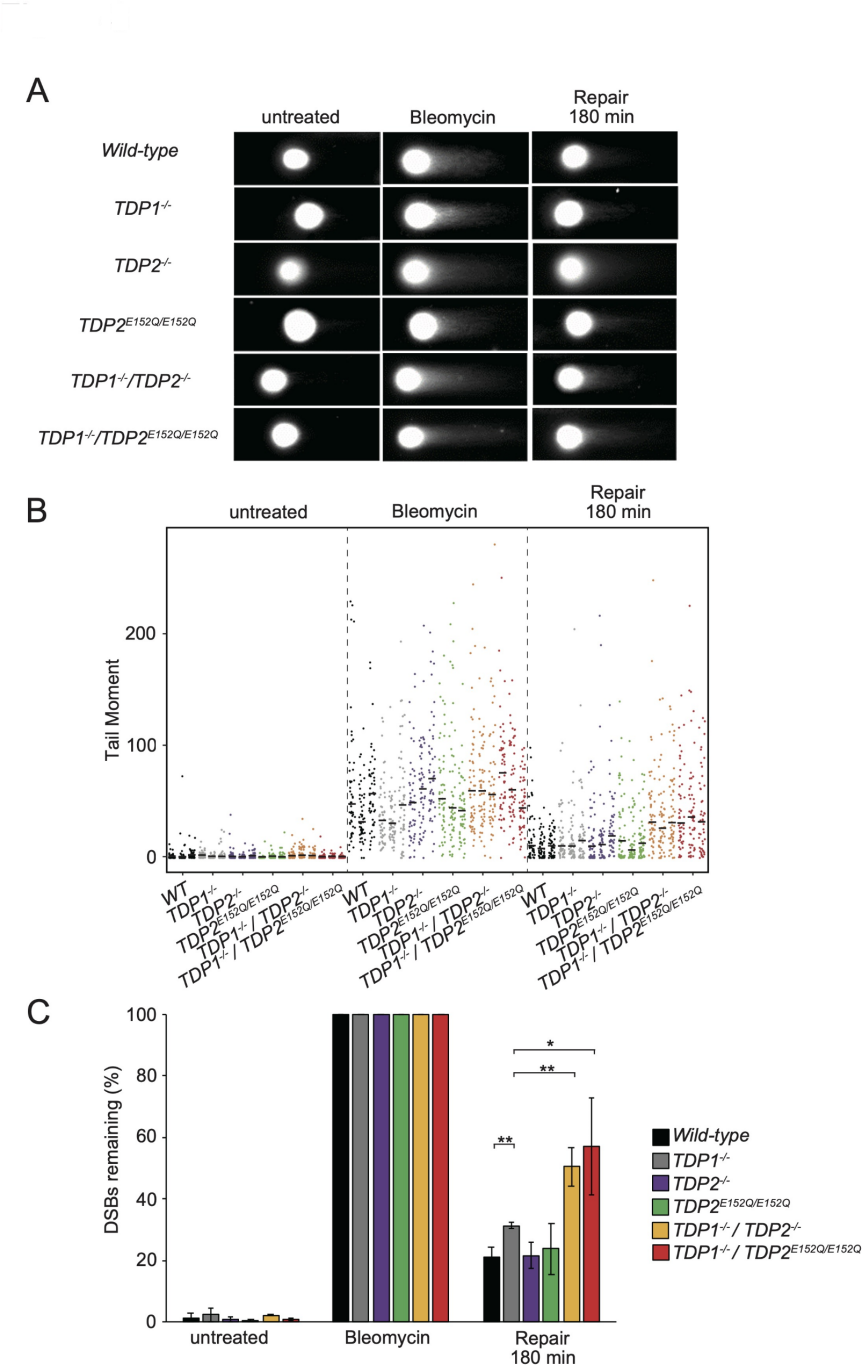
Repair kinetics of DSBs in Bleo-treated cells. **A** Typical neutral comet images. The indicated cells treated with Bleo and not treated with Bleo (control) were analyzed for DSBs using neutral comet assays as described in the legend of Fig. 2A. **B** Tail moments were quantified as described in the legend of Fig. 2B. **C** DSBs remaining was calculated as described in the legend of Fig. 2C. Significant differences were identified using ANOVA and Student’s t-test: **p < 0.01, *p < 0.05. Median tail moments before standardization are shown in Fig. S1B

Next, we examined the repair kinetics of Bleo-induced DSBs in *wild-type*, *TDP1*^*−/−*^, *TDP2*^*−/−*^, *TDP2*^*E152Q/E152Q*^, *TDP1*^*−/−*^*/TDP2*^*−/−*^, and *TDP1*^*−/−*^*/TDP2*^*E152Q/E152Q*^ cells. These cells were treated with 400 µg/mL Bleo for 30 min and allowed to recover in a drug-free medium for 180 min. Figure 3A shows typical images from a neutral comet assay. In all cell types, Bleo treatment caused an increase in the tail moment (Fig. 3B). Due to the observed variation in tail moments among mutants following Bleo treatment (Supplementary Table 2, Fig. S1B), we applied data standardization for the analysis of DSB repair capacity (Fig. 3C). At 180 min after Bleo treatment, significant differences in the remaining DSBs were observed among *wild-type*,* TDP1*^*−/−*^, *TDP1*^*−/−*^*/TDP2*^*−/−*^, and *TDP1*^*−/−*^*/TDP2*^*E152Q/E152Q*^ cells (*p* < 0.005, ANOVA). Compared with *TDP1*^*−/−*^cells, *TDP*^*−/−*^*/TDP2*^*−/−*^ cells exhibited a significant delay in DSB repair (t-test, Fig. 3C). Thus, TDP1 and TDP2 perform overlapping functions in the repair of Bleo-induced DSBs.

In addition, *TDP1*^*−/−*^*/TDP2*^*E152Q/E152Q*^ cells exhibited a significant delay in DSB repair compared with *TDP1*^*−/−*^ cells (t-test, Fig. 3C). Therefore, Glu152 of TDP2 plays a pivotal role in the repair of Bleo-induced DSBs in the absence of TDP1.

Bleo predominantly induces blunt-ended and 5′-staggered 3′-PG DSBs. Previous studies have demonstrated TDP1’s role in removing 3′-PG from DNA in biochemical experiments [44–47]. However, whether TDP2 can also remove 3′-PG remains unclear. Thus, we conducted biochemical experiments using recombinant human TDP2 and a single-stranded DNA substrate harboring a 3′-PG end (Fig. S2A). We first optimized buffer conditions for TDP1 activity, identifying Buffer A—50 mM Tris-HCl (pH 7.5), 1 mM DTT, 25 mM KCl, and 0.1 mg/ml BSA—as most effective for 3′-PG removal (Fig. S2B). We then tested TDP2 under the same conditions as TDP1; however, we did not detect any 3′-PG removal (Fig. S2C). Based on these biochemical findings, we were unable to conclusively determine whether TDP2 is involved in 3′-PG removal.

## Discussion

In this study, we confirmed that both NHEJ and HR facilitated DSB repair induced by X-rays and Bleo. Furthermore, we identified *TDP2* as a susceptible gene following treatment with radiomimetic drugs, but not with X-rays, in the absence of TDP1.

This study provides significant insights into the DNA repair pathways in response to Bleo-induced DNA damage. Our results demonstrate that NHEJ is the predominant pathway contributing to the repair of Bleo-induced DSBs. This conclusion is supported by the pronounced sensitivity of NHEJ-deficient cells (*LIG4*^*−/−/−*^, *DNA-PKcs*^*−/−*^, and *53BP1*^*−/−*^) to Bleo treatment, as shown in Fig. 1, underscoring the critical role of NHEJ in DNA repair following exposure to Bleo. Moreover, the greater sensitivity of *RAD54*^*−/−*^*/LIG4*^*−/−/−*^ cells compared with each single mutant cell indicates that HR also plays a significant role in repairing Bleo-induced DNA damage (Fig. 1). This additive effect suggests that HR functions alongside NHEJ to facilitate DSB repair, highlighting the collaborative contributions of these pathways to cell survival after Bleo treatment.

In addition to the roles of NHEJ and HR, our findings reveal a crucial contribution of TDP1 to the repair of Bleo-induced DSBs. *TDP1*^*−/−*^ cells, but not *TDP2*^*−/−*^ cells, exhibited increased sensitivity to Bleo and a delay in DSB repair, indicating that TDP1 is involved in the repair of DSBs generated by Bleo treatment in *wild-type* cells. Furthermore, the *TDP1*^*−/−*^*/TDP2*^*−/−*^ cells showed even greater sensitivity to Bleo and a more pronounced delay in DSB repair compared with *TDP1*^*−/−*^ cells alone. This suggests that in the absence of TDP1, TDP2, along with NHEJ and HR, contributes to the repair of Bleo-induced damage. TDP2 may act as a backup mechanism for processing DNA termini when TDP1 is absent. Overall, these findings indicate that Bleo-induced DSB ends contain lesions recognized by TDP1 and TDP2. Once these lesions are removed by TDP1 or TDP2, the resulting DSBs may subsequently be repaired by HR or NHEJ.

We found that *TDP1*^*−/−*^*/TDP2*^*−/−*^ cells exhibited greater sensitivity to Bleo than *TDP1*^*−/−*^ or *TDP2*^*−/−*^ single mutants, but not to X-rays. This discrepancy may be explained by the distinct DNA damage profiles induced by Bleo versus IR. Unlike IR, Bleo predominantly generates 3′-PG ends at DNA breaks, requiring specific repair mechanisms [5]. In addition, TDP1 processes 3′-PG [44–46, 48, 49] and suppresses the misjoining of radiomimetic DSBs [50]. Consequently, we sought to determine whether TDP2 is directly involved in removing 3′-PG. However, it is not known that any method exists for specifically labeling Bleo-induced 3′-PG, making direct analysis of 3′-PG removal at the cellular level unfeasible. Therefore, we carried out biochemical experiments using recombinant human TDP2 and a DNA substrate harboring a 3′-PG end (Fig. S2A). Under these conditions, TDP1 processed 3′-PG in vitro (Fig. S2B); however, TDP2 failed to do so (Fig. S2C). These results suggest several possibilities. First, TDP2 may simply lack 3′-PG removal activity in vitro, instead participating in the repair of other minor lesions, such as DPCs. Bleo is activated upon binding to oxygen molecules, potentially leading to DNA–protein crosslinking. This minor form of damage could be a target for TDP2. Second, the optimal buffer conditions for TDP1 and TDP2 may differ. This can be explained by the fact that TDP2’s 3′-TDP activity requires a divalent metal ion, whereas TDP1’s does not [24]. Therefore, we hypothesized that TDP2’s 3′-PG processing activity also depends on a divalent metal ion. However, TDP2 did not exhibit 3′-PG processing activity in buffers containing Mg²⁺ (buffer B, C, D, F, G, H) (Fig. S2C), suggesting that the Mg²⁺ concentration or the pH may not have been optimized for this reaction. Third, TDP2 may require additional cofactors to act on 3′-PG; for example, a chaperon may help TDP2 access to the 3′-PG site or promote the catalytic activity of TDP2 for 3′-PG in *TDP1*^*−/−*^ cells. In the second and third scenarios, TDP2 could potentially remove 3′-PG damage in the absence of TDP1 in cells, functioning as a backup mechanism for this particular lesion. By contrast, IR-induced breaks comprise diverse and complex lesions that are repaired by a broader set of enzymes involved in HR or NHEJ, making TDP2’s specialized function less critical for IR-induced damages. Nonetheless, these possibilities remain speculative, and further studies are needed to clarify the precise role of TDP2 in repairing 3′-PG damage.

We previously suggested that the inhibition of TDP2 combined with a TOP1 inhibitor and chain-terminating nucleoside analogs may be an effective strategy for tumor treatment [30]. We also suggested that enhancing Mg^2+^ chelating efficiency or breaking the association between Glu152 and Mg^2+^ may lead to the development of TDP2 inhibitors with increased efficacy [24]. TDP2 can repair DSBs induced by radiomimetic drugs (Fig. 3). Therefore, TDP2 inhibition combined with a TOP1 inhibitor and radiomimetic drugs results in cancer cell sensitization. Based on the higher sensitivity of *TDP1*^*−/−*^*/TDP2*^*E152Q/E152Q*^ cells to radiomimetic drugs than single mutants, breaking the bond between TDP2 and divalent metal ions may be important in radiomimetic drug therapy. Development of improved TDP2 inhibitors is anticipated in the future.

The TK6 mutant panel profile enabled us to identify important genes to be targeted for drug development. For example, *RAD54*^*−/−*^*/LIG4*^*−/−/−*^ cells are the most sensitive mutant cells to X-rays and radiomimetic drugs, which confirms the synergistic combination of radiomimetic drugs and DSB repair inhibitors. Since numerous genes associated with DNA damage and repair responses are frequently altered in human cancers, identifying the faulty genes within each tumor cell is essential to enhance the efficacy of anticancer drugs. By selecting drugs that trigger DNA damage repair specific to the defective gene, we can expect a more potent cell-killing effect. The sensitivity profile of the TK6 mutants established in this study offers a logical method to assess the significance of individual repair pathways and genes as potential targets in chemotherapy.

The combination of TDP inhibitors with radiomimetic drugs such as Bleo shows promise as a therapeutic strategy but also presents several challenges. A primary concern is off-target toxicity: while TDP inhibitors enhance the efficacy of radiomimetic drugs by impairing specific DNA repair pathways, they may inadvertently disrupt other cellular processes, resulting in unintended harm to non-target cells. Moreover, resistance mechanisms can emerge during prolonged treatment. Cancer cells may acquire mutations or upregulate alternative repair pathways, such as NHEJ or HR, thereby diminishing the therapy’s effectiveness. To address these limitations, the development of biomarkers for predicting and monitoring treatment responses is crucial. In addition, combination regimens must be carefully optimized to minimize off-target effects and delay resistance, for example by employing intermittent dosing schedules or incorporating agents that target alternative repair pathways.

## Conclusions

In this study, we identified *TDP2* as a susceptible gene in the absence of *TDP1* to target cancers with radiomimetic drugs. TDP2 is important for the repair of radiomimetic drug-induced DSBs in the absence of TDP1. Elucidation of the TDP2-dependent repair pathway for DSBs induced by radiomimetic drugs revealed that the combination of radiomimetic drugs, TDP1 inhibitors, and TDP2 inhibitors can be used for cancer treatment. Further studies are needed to elucidate the detailed repair mechanism of TDP2 for DNA damage induced by radiomimetic drugs.

## Supplementary Information

Supplementary Material 1.

Supplementary Material 2.

Supplementary Material 3.

Supplementary Material 4.

## Data Availability

The data generated and analyzed during the current study are available from the corresponding author on reasonable request.

## Abbreviations

BER: Base excision repair
Bleo: Bleomycin
CAL: Calicheamicin
CPT: Camptothecin
DPCs: DNA-protein crosslinks
DSBs: DNA double-strand breaks
HR: Homologous recombination
IR: Ionizing radiation
MMR: Mismatch repair
NER: Nucleotide excision repair
NHEJ: Nonhomologous end joining
3′-PG: 3′-phosphoglycolate
SSBs: DNA single-strand breaks
TDP: Tyrosyl-DNA phosphodiesterase
TLS: Translesion DNA synthesis
TOP: Topoisomerase
TOP1ccs: TOP1-DNA cleavage complexes
TOP2ccs: TOP2-DNA cleavage complexes
