# Supplementary figures and images for: Role of TDP2 in the repair of DNA damage induced by the radiomimetic drug Bleomycin
Source: Genes Environ. 2025 Mar 28;47:7. doi: 10.1186/s41021-025-00329-9 (PMC11954286; doi:10.1186/s41021-025-00329-9)

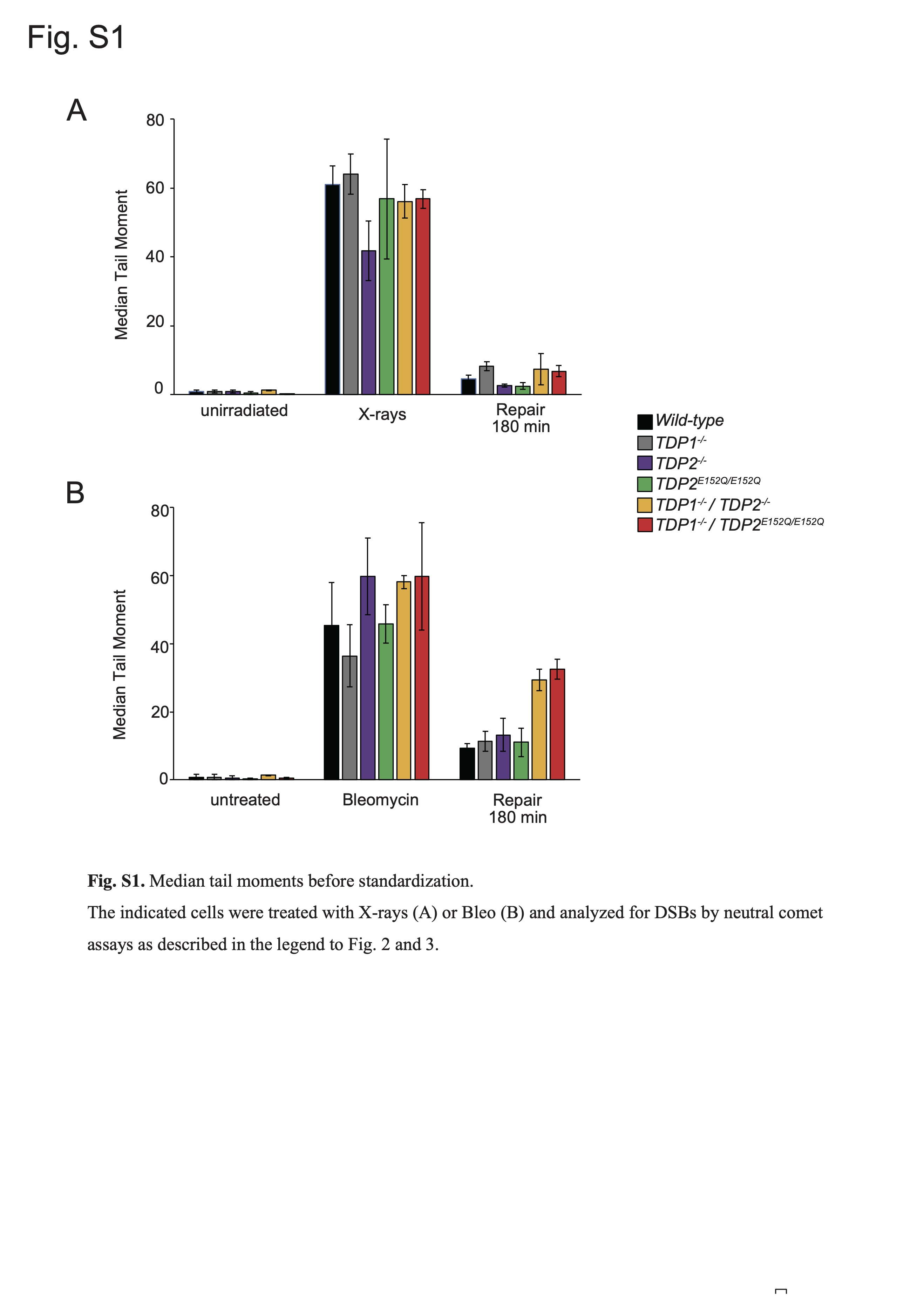

Supplement: Supplementary file 1 — Supplementary Material 1. [file 41021_2025_329_MOESM1_ESM.jpg]

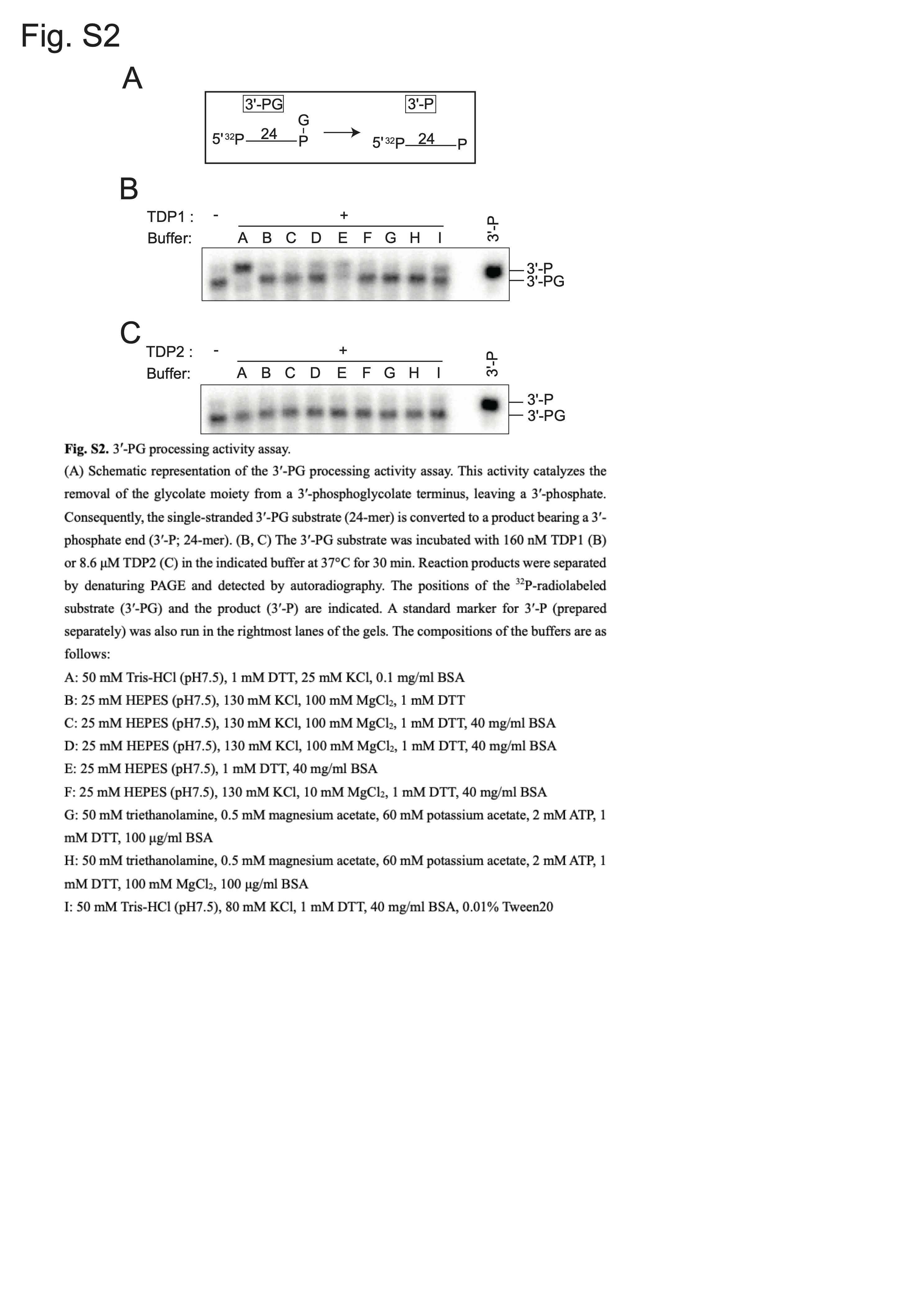

Supplement: Supplementary file 4 — Supplementary Material 4. [file 41021_2025_329_MOESM4_ESM.jpg]
